# Epidermoid Cysts in an Intrapancreatic Accessory Spleen Mimicking Pancreatic Neoplasms: Two Case Reports and a Literature Review of Japanese Cases

**DOI:** 10.70352/scrj.cr.26-0039

**Published:** 2026-05-26

**Authors:** Xiufeng Li, Qingjiang Hu, Yu Takashi, Koto Kawata, Shohei Shibuta, Yuki Ando, Hajime Otsu, Yusuke Yonemura, Taro Tobo, Koshi Mimori

**Affiliations:** 1 Department of Surgery, Kyushu University Beppu Hospital, Beppu, Oita, Japan; 2 Precision Pathology Diagnosis Center, Weifang People’s Hospital, Weifang, Shandong, China; 3 Department of Clinical Laboratory Medicine, Kyushu University Beppu Hospital, Beppu, Oita, Japan

**Keywords:** epidermoid cyst, intrapancreatic accessory spleen, pancreatic cystic neoplasm, diagnostic challenge, clinical decision-making

## Abstract

**INTRODUCTION:**

Epidermoid cyst(s) arising in an intrapancreatic accessory spleen (ECIPAS) are rare benign lesions that often mimic pancreatic cystic neoplasms on imaging studies. Owing to their rarity and nonspecific radiological features, ECIPAS are frequently misdiagnosed preoperatively, leading to unnecessary pancreatic resections.

**CASE PRESENTATION:**

We report 2 asymptomatic middle-aged women who were incidentally found to have cystic lesions in the tail of the pancreas during routine imaging examinations. Contrast-enhanced CT and MRI demonstrated well-demarcated cystic lesions with low signal intensity on T1-weighted images and high signal intensity on T2-weighted images. In both cases, mucinous cystic neoplasms or intraductal papillary mucinous neoplasms were suspected, prompting distal pancreatectomies, with a concomitant splenectomy in 1 patient. Histopathological examination revealed cysts lined predominantly by stratified squamous epithelium and surrounded by normal splenic tissue, confirming the diagnosis of ECIPAS. In addition, a review of the Japanese literature identified 52 reported cases of ECIPAS; most patients were asymptomatic, with lesions predominantly located in the pancreatic tail and frequently associated with elevated serum carbohydrate antigen 19-9 levels, although normal levels are also common.

**CONCLUSIONS:**

Recognizing ECIPAS may support more cautious clinical decision-making and help surgeons consider less invasive management strategies, including spleen-preserving distal pancreatectomy when appropriate.

## Abbreviations

CA19-9
: carbohydrate antigen 19-9
CEA
: carcinoembryonic antigen
ECIPAS
: epidermoid cyst(s) arising in an intrapancreatic accessory spleen
IPMN
: intraductal papillary mucinous neoplasm
MCN
: mucinous cystic neoplasm

## INTRODUCTION

ECIPAS are rare benign cystic lesions of the pancreas. An accessory spleen is present in approximately 10%–30% of the general population, most commonly located near the splenic hilum^1)^; however, intrapancreatic accessory spleens are relatively uncommon.^2)^ Additionally, the formation of an epidermoid cyst within an intrapancreatic accessory spleen is extremely rare,^3)^ accounting for only a small fraction of pancreatic cystic lesions.

Clinically, ECIPAS pose a diagnostic challenge because the imaging characteristics often overlap with those of pancreatic cystic neoplasms,^4,5)^ such as MCNs and IPMNs. On CT and MRI, ECIPAS typically present as well-circumscribed cystic lesions in the pancreatic tail and frequently manifest nonspecific signal intensities. In addition, serum CA19-9 levels may be elevated in some cases, further complicating the preoperative differentiation of ECIPAS from malignant or potentially malignant pancreatic tumors. Consequently, many patients undergo surgical resection for suspected pancreatic neoplasms, and the definitive diagnosis of ECIPAS is often established only after histopathological evaluation.

Given the benign nature of ECIPAS, an accurate preoperative identification is clinically important for avoiding unnecessary pancreatic resection. However, owing to its rarity, ECIPAS is often overlooked in daily clinical practice when considering the differential diagnosis based on preoperative findings. In this report, we describe 2 cases of ECIPAS that were preoperatively misdiagnosed as pancreatic cystic neoplasms and subsequently treated surgically. In addition, we review the Japanese literature to summarize the clinicopathological characteristics of ECIPAS and discuss the key preoperative features that may aid in its differential diagnosis.

## CASE PRESENTATION

### Case 1

A 51-year-old woman was incidentally found to have a cystic lesion in the tail of her pancreas during an abdominal ultrasonography examination performed as part of a routine health check-up. Her past history was negative for hypertension, diabetes mellitus, trauma, surgery, or pancreatitis, and there was no family history of genetic diseases. She was asymptomatic on presentation. Her physical examination was unremarkable. Laboratory tests, including tumor markers, were within normal limits, with a CEA level of 1.1 ng/mL (normal range, 0–5 ng/mL) and a CA19-9 level of 7.7 U/mL (normal range, <37 U/mL).

**Fig. 1:**
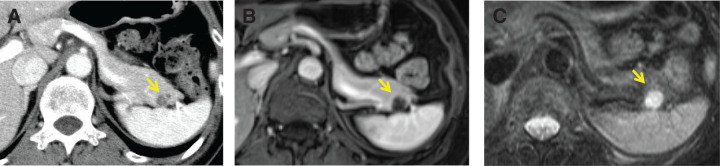
Imaging manifestations of pancreatic lesions (as indicated by the yellow arrow) in Case 1. (**A**) Abdominal CT shows the tail of the pancreas and the wall of the cyst, which is partially enhanced. (**B**) MRI reveals a cystic lesion with low signal intensity on T1-weighted images. (**C**) MRI reveals a cystic lesion with high signal intensity on T2-weighted images.

Abdominal CT revealed a 15-mm cystic mass in the tail of the pancreas with partial enhancement of the cyst wall (**Fig. 1A**). MRI showed a cystic lesion with low signal intensity on T1-weighted images (**Fig. 1B**) and high signal intensity on T2-weighted images (**Fig. 1C**), with no other abnormalities. The lesion was well-circumscribed. No definite mural nodules or internal septations were clearly identified on imaging. Communication with the main pancreatic duct was not evident. The lesion and spleen were visualized on the same imaging phase; however, the accessory splenic component was not clearly identifiable, which limited preoperative recognition. Based on these findings, an MCN or IPMN was suspected, and a spleen-preserving distal pancreatectomy was performed. The lesion was small (15 mm) and showed no apparent involvement of the splenic vessels or parenchyma, allowing preservation of the spleen.

**Fig. 2:**
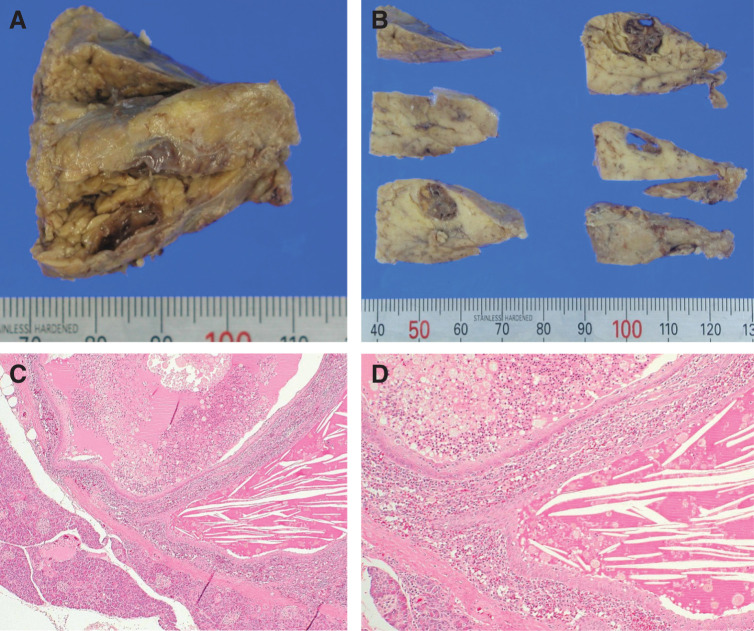
The gross and histopathological manifestations of the lesion in Case 1. (**A**) The lesion is located within the pancreatic tissue. (**B**) The lesion is cystic, with a dark red cut surface and multiple lobes. (**C**) The cystic cavities contain amorphous eosinophilic material and cholesterol clefts, with intervening splenic tissue between the cavities (HE staining, magnification ×40). (**D**) The lining of the cyst is composed of stratified squamous epithelium (HE staining, magnification ×100). HE, hematoxylin and eosin

Gross examination of the resected specimen revealed a well-circumscribed, encapsulated cystic lesion within the pancreatic tissue (**Fig. 2A**). The cut surface was dark red, cystic, and multilocular (**Fig. 2B**). Microscopic examination showed that the cyst was separated from the surrounding pancreatic tissue by a fibrous capsule (**Fig. 2C**). The cyst consisted of cavities of various sizes containing amorphous eosinophilic material and cholesterol clefts, with thin layers of splenic tissue interposed between the cavities (**Fig. 2D**). The cyst was lined predominantly by stratified multilayered squamous epithelium without keratinization. These findings were consistent with ECIPAS.

### Case 2

A 50-year-old woman was incidentally found to have cystic lesions in the tail of her pancreas during an abdominal US examination at a routine physical check-up. Her past history was negative for hypertension, diabetes mellitus, trauma, surgery, or pancreatitis, and there was no relevant family history. She was asymptomatic at presentation. Laboratory tests, including tumor markers, were within normal limits, with a CEA level of 1.3 ng/mL and a CA19-9 level of 15.4 U/mL.

**Fig. 3:**
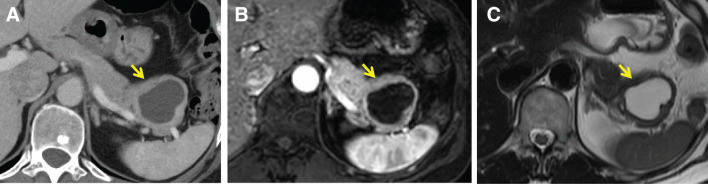
Imaging manifestations of the pancreatic lesions (as indicated by the yellow arrow) in Case 2. (**A**) CT shows the tail of the pancreas and the cyst wall. (**B**) MRI reveals a cystic lesion with low signal intensity on T1-weighted images. (**C**) MRI reveals a cystic lesion with high signal intensity on T2-weighted images.

Abdominal CT revealed a 42-mm cystic mass in the pancreatic tail with suspected marginal calcifications (**Fig. 3A**). MRI demonstrated a cystic lesion with low signal intensity on T1-weighted images (**Fig. 3B**) and high signal intensity on T2-weighted images (**Fig. 3C**), with no other abnormalities. The lesion was well-circumscribed with a relatively thickened cyst wall. No definite mural nodules or internal septations were clearly identified. There was no apparent communication with the pancreatic duct. The lesion appeared closely associated with the spleen; however, the accessory splenic component was not clearly identifiable, which limited preoperative recognition. An MCN was clinically suspected, and a distal pancreatectomy with splenectomy was performed. The lesion was relatively large (42 mm) and was closely adjacent to the spleen, making splenectomy appropriate from a surgical safety perspective.

**Fig. 4:**
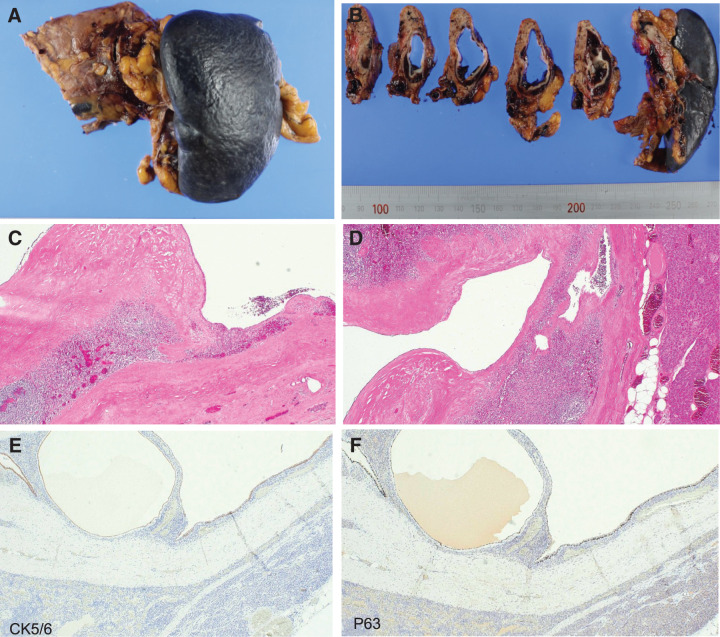
Gross and histopathological manifestations of the lesion in Case 2. (**A**) The lesion is located in the tail of the pancreas. (**B**) The lesion is a unilocular cyst containing brownish liquid and has a capsule. (**C**) The inner lining of the cyst is lined by squamous and cuboidal epithelium, and the outer side of the cyst wall shows splenic tissue (HE staining, magnification 40×). (**D**) Within the surrounding splenic tissue, some smaller cysts are also seen that are lined internally with stratified squamous epithelium (HE staining, magnification ×40). (**E**) The epithelium lining the cyst wall is positive for CK5/6 expression (IHC staining, magnification ×40). (**F**) The epithelium lining the cyst wall is positive for p63 expression (IHC staining, magnification ×40). CK5/6, cytokeratin 5/6; HE, hematoxylin and eosin; IHC, immunohistochemical

Gross examination of the resected specimen revealed an encapsulated, unilocular cystic lesion containing brownish fluid (**Fig. 4A** and **4B**). Microscopically, the cyst showed a clear boundary from the surrounding tissue. The cyst wall consisted of fibrous tissue of variable thickness (**Fig. 4C**), with small duct-like structures observed near the pancreatic side of the cyst wall. Splenic tissue was present on the outer side of the cyst wall, with pancreatic tissue located further outside the splenic tissue (**Fig. 4D**). The inner surface of the cyst was partially lined by single- or multilayered squamous epithelium with a small component of columnar epithelium (**Fig. 4D**). In some areas, the epithelial lining was partially denuded, and flat cells extending into the stratified squamous epithelium were observed. Additional small cysts lined by stratified squamous epithelium were identified within the splenic tissue (**Fig. 4D**).

Immunohistochemical analysis of the specimens revealed positive staining for cytokeratin, cytokeratin 5/6 (**Fig. 4E**), and tumor protein p63 (**Fig. 4F**) in the epithelium lining the cyst, whereas S-100 and cluster of differentiation 34 proteins were negative. Silver staining clearly demonstrated pancreatic tissue surrounding splenic tissue and around the cyst walls. These findings confirmed the diagnosis of ECIPAS.

### Review of previously reported cases

To clarify the clinicopathological characteristics of epidermoid cysts arising in an intrapancreatic accessory spleen, we performed a narrative review of 37 articles reporting 52 Japanese cases, including the 2 present cases, which were published between 1981 and 2024 (**Table 1**).^6–42)^ A literature search was conducted using PubMed with the term “epidermoid cyst in an intrapancreatic accessory spleen,” including reports published between 1981 and 2024 in English and Japanese. This review focused on Japanese cases, which represent the majority of reported ECIPAS cases, providing a more consistent and clinically relevant summary that complements existing international reports. Most patients were asymptomatic, whereas abdominal pain or discomfort was reported in a minority of cases. The male-to-female ratio was 23:29, and the mean age of the patients was 49.3 years (range, 32–73 years). Serum CEA levels were normal in all cases, while CA19-9 levels were elevated in 25 (53%) patients and subsequently decreased to the normal range after surgical resection. All lesions were located in the pancreatic tail, with cystic diameters ranging from 1.0 to 13.4 cm (mean, 3.6 cm). Only 15% of cases were diagnosed or suspected preoperatively. Surgical resection was performed in all cases, and the most common procedure was a distal pancreatectomy with or without splenectomy.

**Table 1:** Clinical and pathological characteristics of cases of ECIPAS in Japan

Factors	Number/total (%)
Age (years)	
<40	14/52 (27)
40–65	33/52 (63)
>65	5/52 (10)
Gender	
Male	23/52 (44)
Female	29/52 (56)
Location of the tumor	
Tail	52/52 (100)
Not tail	0
Symptoms	
Abdominal pain	9/52 (17)
Epigastric discomfort	1/52 (2)
Back pain/discomfort	3/52 (6)
Diarrhea	1/52 (2)
None	38/52 (73)
Preoperative serum CA19-9 (U/mL)	
High	25/47 (53)
Normal	22/47 (47)
T1 MRI signal	
High	2/19 (10)
Low	14/19 (74)
T2 MRI signal	
High	12/20 (60)
Low	1/20 (5)
Maximum diameter (cm)	
≤3	29/52 (56)
>3	23/52 (44)
Locularity	
Unilocular	23/52 (44)
Multilocular	29/52 (56)
Pre-treatment diagnosis	
ECIPAS	6/40 (15)
MCN	17/40 (43)
PCT	12/40 (30)
PC	2/40 (5)
IPMN + SPT + PP	3/40 (7)
Surgically resected	
DP	21/51 (41)
DPS	30/51 (59)

CA19-9, carbohydrate antigen 19-9; DP, distal pancreas; DPS, distal pancreas and spleen; ECIPAS, epidermoid cyst in intrapancreatic accessory spleen; IPMN, intracavitary papillary mucinous tumor; MCN, mucinous cystic tumor; PC, pancreatic cyst; PCT, pancreatic cystic tumor; PP, pancreatic pseudocyst; SPT, solid papillary tumor

## DISCUSSION

ECIPAS represents a rare benign entity that is frequently misdiagnosed as a pancreatic cystic neoplasm.^6)^ This report of our cases, together with our literature review, highlights several factors that contributed to the difficulty of achieving an accurate preoperative diagnosis.

First, our review of previously reported cases found that the characteristic manifestations of ECIPAS included a slight tendency to affect more female than male patients,^43)^ occurrence in middle-aged patients, absence of symptoms, predominant location in the pancreatic tail,^3)^ and unilocular morphology.^43)^ Such characteristics are not specific to ECIPAS and therefore provide limited diagnostic value.

Second, the imaging features of ECIPAS are nonspecific and overlap considerably with those of pancreatic cystic neoplasms, such as MCN and IPMN. On CT and MRI, ECIPAS typically appear as well-demarcated cystic lesions in the pancreatic tail with low signal intensity on T1-weighted images and high signal intensity on T2-weighted images.^44)^ These findings are common among various cystic lesions of the pancreas and do not allow reliable differentiation. In addition, mural enhancement or septations may be observed, further raising suspicion for neoplastic lesions.

A structured comparison with other cystic pancreatic lesions may aid in the differential diagnosis. MCN typically presents in middle-aged women as a cystic lesion with a thick wall, septations, and occasional mural nodules, without ductal communication. IPMN is characterized by communication with the pancreatic duct and often shows ductal dilation or mural nodules. In contrast, ECIPAS usually appears as a cystic lesion in the pancreatic tail without ductal communication and tends to lack prominent neoplastic features. Importantly, the presence of adjacent tissue with imaging characteristics similar to the spleen may serve as a key diagnostic clue. These differences may explain why our cases were initially suspected to be MCN or IPMN.

Third, serum tumor marker levels, particularly CA19-9, may be elevated in patients with ECIPAS.^43)^ As found in this review, more than half of the reported cases showed elevated CA19-9 levels, which returned to the normal range after surgical resection. Immunohistochemical studies have revealed that the epithelium lining the cyst expresses CA19-9,^7,8)^ suggesting that elevated serum CA19-9 levels originate from the cyst itself rather than from malignant transformation. However, a substantial proportion of patients, including our 2 cases, have normal CA19-9 levels. Therefore, CA19-9 should be considered a supportive finding rather than a diagnostic trigger in the evaluation of ECIPAS.

Finally, the rarity of ECIPAS and limited awareness among clinicians contribute substantially to diagnostic difficulty. Intrapancreatic accessory spleens themselves are uncommon, and cystic transformation within accessory splenic tissue is even rarer. Consequently, ECIPAS is not routinely included in the differential diagnosis of cystic pancreatic lesions, particularly when the typical imaging features of accessory spleens are absent. In addition, the natural history of ECIPAS remains unclear, as most lesions are resected shortly after detection.

From a practical clinical perspective, although a definitive preoperative diagnosis of ECIPAS remains difficult, certain imaging findings may raise suspicion for this entity and influence management strategies. In particular, the presence of a cystic lesion in the pancreatic tail, combined with adjacent or surrounding tissue showing imaging characteristics similar to those of the spleen on contrast-enhanced CT or MRI, should prompt consideration of ECIPAS. Additional supportive findings may include signal intensity matching that of splenic tissue on diffusion-weighted imaging or superparamagnetic iron oxide–enhanced MRI. While these features are not sufficient to establish a definitive diagnosis, recognizing them may help clinicians include ECIPAS in the differential diagnosis and adopt a more cautious approach to treatment planning, rather than proceeding directly to surgery based solely on the suspicion of mucinous neoplasms.

Given the benign nature of ECIPAS, avoiding unnecessary resections of the pancreas is clinically important. However, complete avoidance of surgery is not always feasible because malignancy cannot be confidently excluded preoperatively. Therefore, improving diagnostic suspicion is essential to guide more appropriate management strategies. Awareness of the characteristic clinical patterns of ECIPAS is essential; ECIPAS typically occur in asymptomatic middle-aged patients and is almost exclusively located in the pancreatic tail. In addition, careful evaluation of imaging studies may provide useful clues, particularly when findings suggest similarity to splenic tissue.^9,45,46)^

Endoscopic US-guided fine-needle aspiration may also aid in the diagnosis by excluding malignant features^47)^; however, the diagnostic yield of fine-needle aspiration for ECIPAS remains limited, and false-negative results for malignancy cannot be completely ruled out. Endoscopic US was not performed in our cases, reflecting the clinical scenario in which ECIPAS is often not suspected preoperatively and surgical resection is selected based on presumed mucinous neoplasms.

Ultimately, when ECIPAS is suspected but malignancy cannot be confidently excluded, a tailored surgical approach should be considered. Intraoperative frozen-section analysis of the cyst wall or adjacent tissue may help confirm the presence of splenic tissue and avoid extensive resection. Awareness of ECIPAS and inclusion of this entity in the differential diagnosis of pancreatic cystic lesions, particularly those located in the pancreatic tail, are crucial steps toward reducing unnecessary surgical interventions.

## CONCLUSIONS

Epidermoid cysts arising in an intrapancreatic accessory spleen are rare benign lesions that are frequently misdiagnosed as pancreatic cystic neoplasms because of their nonspecific clinical and radiological features. The findings in our cases and literature review indicate that ECIPAS typically occur in asymptomatic patients and are predominantly located in the pancreatic tail, often accompanied by elevated serum CA19-9 levels. Awareness of this entity and careful interpretation of imaging findings are essential for including ECIPAS in the differential diagnosis of pancreatic cystic lesions. Recognizing ECIPAS may support more cautious clinical decision-making and help surgeons consider less invasive management strategies, including spleen-preserving distal pancreatectomy when appropriate.

## DECLARATIONS

### Availability of data and materials

Data will be made available upon reasonable request.
